# Supplementary material for: Substantial lifetime enhancement for Si-based photoanodes enabled by amorphous TiO2 coating with improved stoichiometry
Source: Nat Commun. 2023 Apr 4;14:1865. doi: 10.1038/s41467-023-37154-z (PMC10073107; doi:10.1038/s41467-023-37154-z)
Supplement: Supplementary file 1 — Supplementary Information [file 41467_2023_37154_MOESM1_ESM.pdf]

Supporting information for

Substantial Lifetime Enhancement for Si-Based Photoanodes Enabled by Amorphous TiO_2 Coating with Improved Stoichiometry

Yutao Dong[1], Mehrdad Abbasi[2], Jun Meng[1], Lazarus German[1], Corey Carlos[1], Jun Li[1], Ziyi Zhang[1], Dane Morgan[1], Jinwoo Hwang[2], Xudong Wang[1]*

[1]Department of Materials Science and Engineering, University of Wisconsin-Madison, Madison, WI, 53706, USA

[2]Department of Materials Science and Engineering, The Ohio State University, Columbus, OH 43210, USA

[*] Email: xudong.wang@wisc.edu

Supplementary Figures

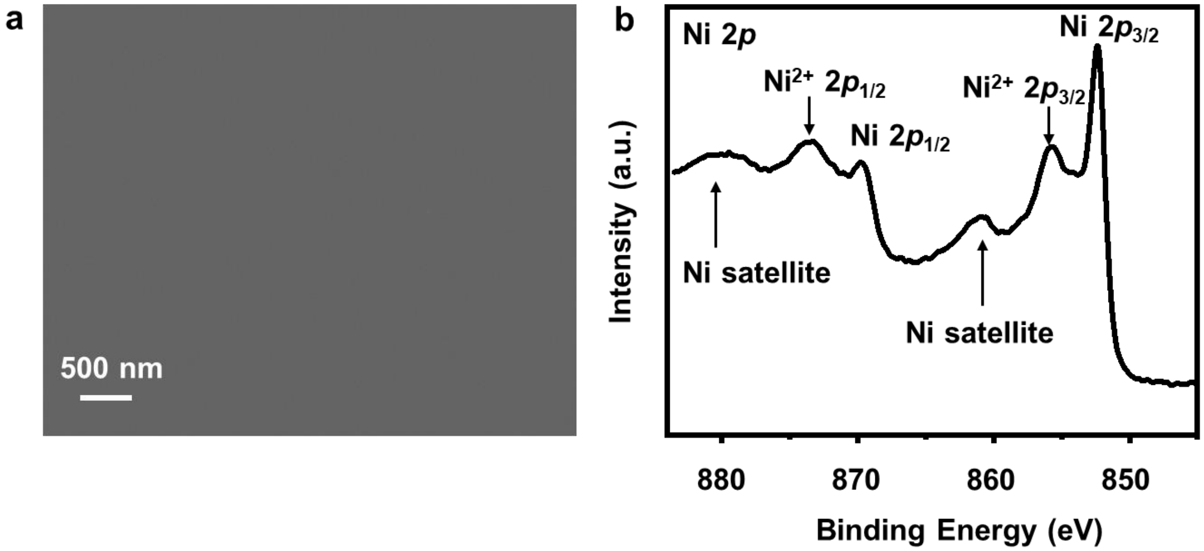

Figure S1. **a** Top-view SEM image of Ni sputtered Si/TiO_2 photoanode. **b** XPS Ni core spectrum of as-prepared Si/TiO_2/Ni photoanode.

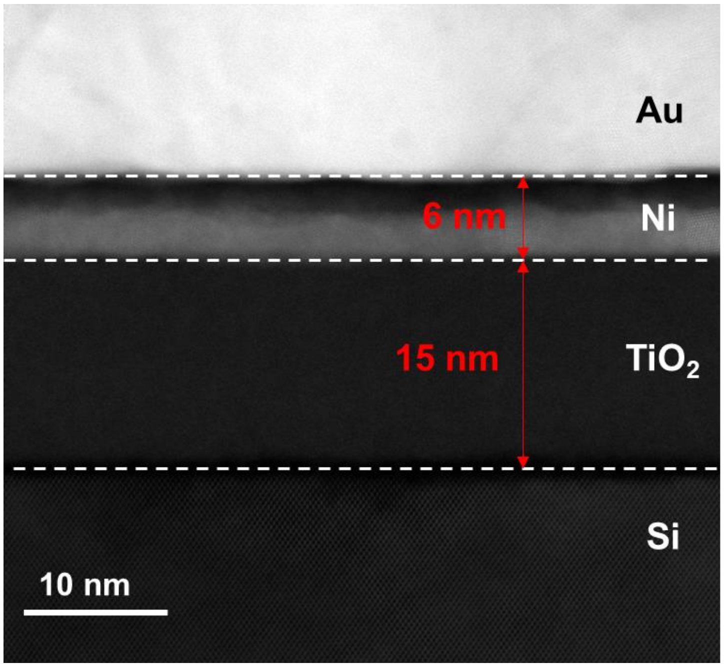

Figure S2. Cross-sectional HR-STEM images of as-prepared ~15 nm Si/TiO_2 with ~6 nm Ni sputtered.

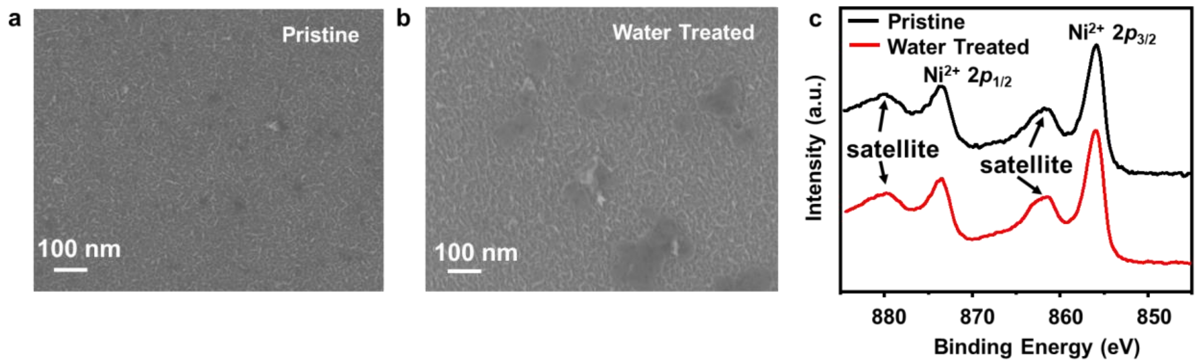

Figure S3. **a**, **b** SEM of Si/TiO_2/Ni after 1h test with pristine **(a)** and water treated **(b)** TiO_2 protection layer. **c** XPS Ni core spectra after 1h test of Si/TiO_2/Ni photoanode with pristine and water treated TiO_2 protection film.

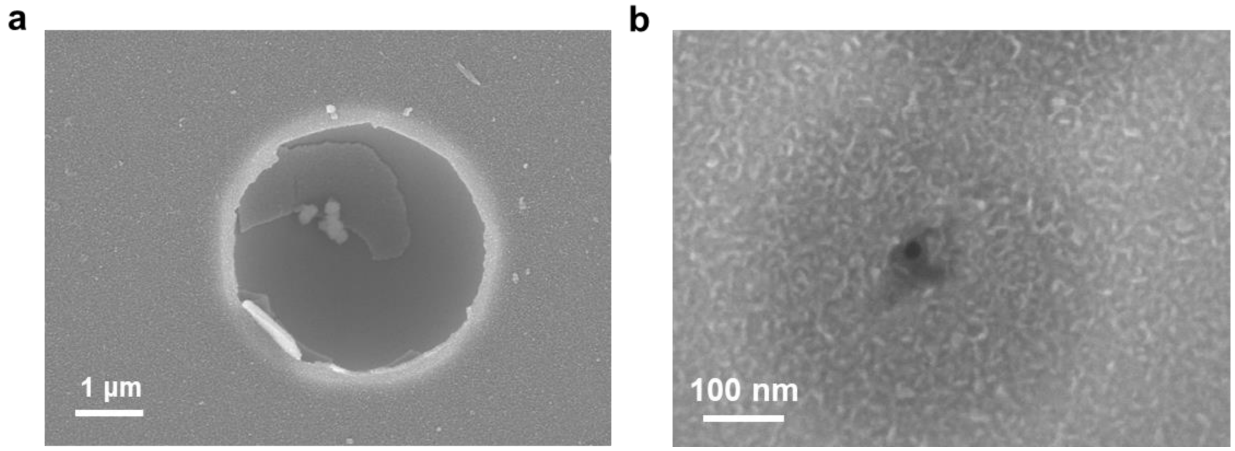

Figure S4. High magnification top-view SEM of pinholes in Si/TiO_2/Ni with pristine TiO_2 thin film during device failure. **(a)** 30h, **(b)** 5 h.

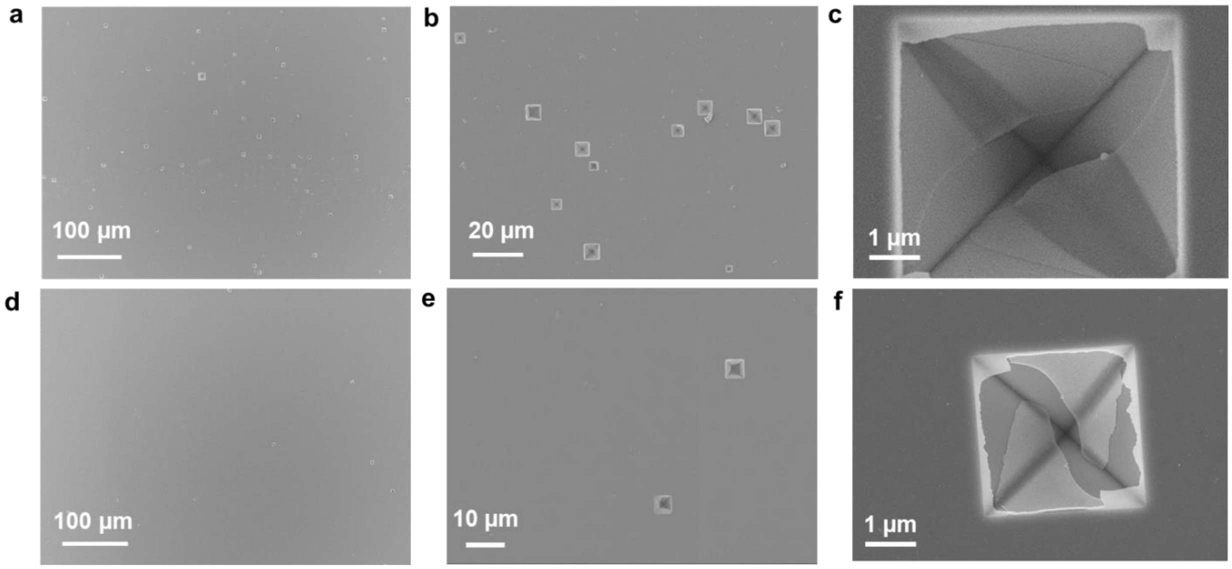

Figure S5. **Chemical stability test of Si/TiO_2 of 1-day immersion in 1M KOH aqueous solution.** Surface morphology of pristine (**a**)-(**c**), and water treated Si/TiO_2 (**d**)-(**f**) under different magnification.

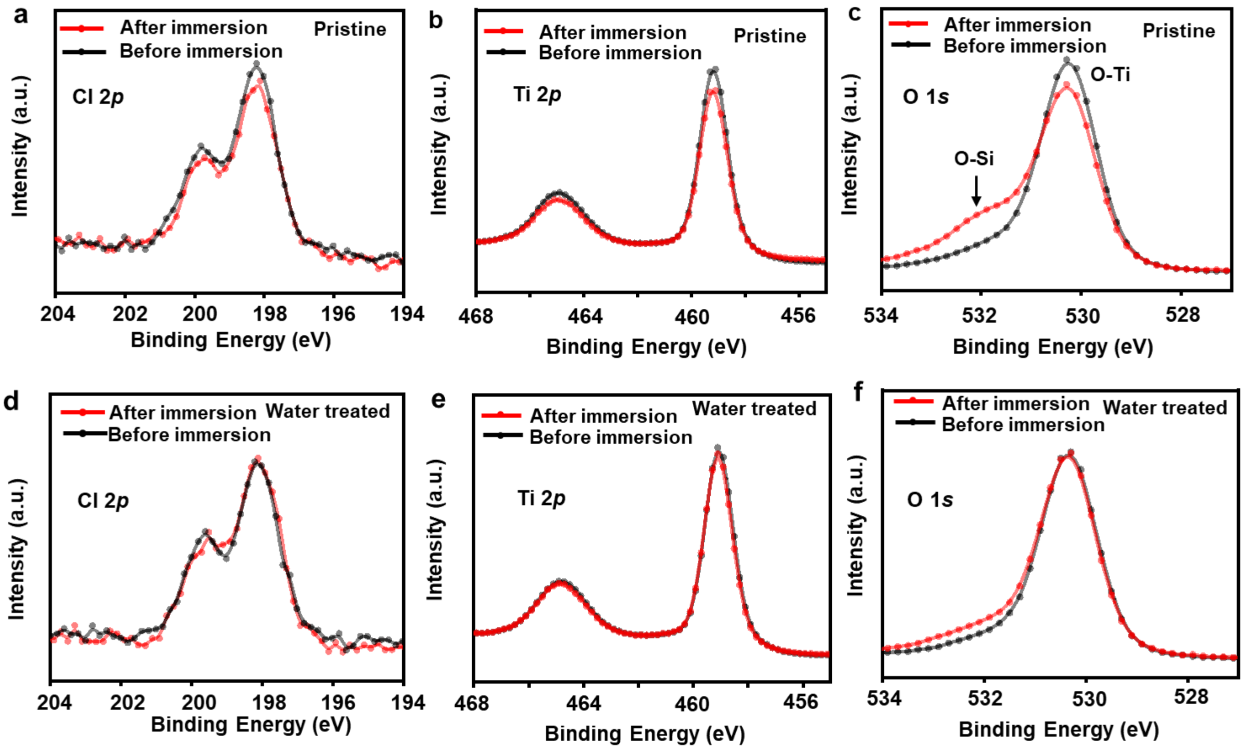

Figure S6. **XPS core spectrum analysis of pristine, water treated Si/TiO_2 wafer before and after immersion in 1M KOH aqueous solution**. **(a)** Cl 2*p*, **(b)** Ti 2*p*, **(c)** O 1*s* of Pristine TiO_2. **(d)** Cl 2*p*, **(e)** Ti 2*p*, **(f)** O 1*s* of water treated TiO_2.

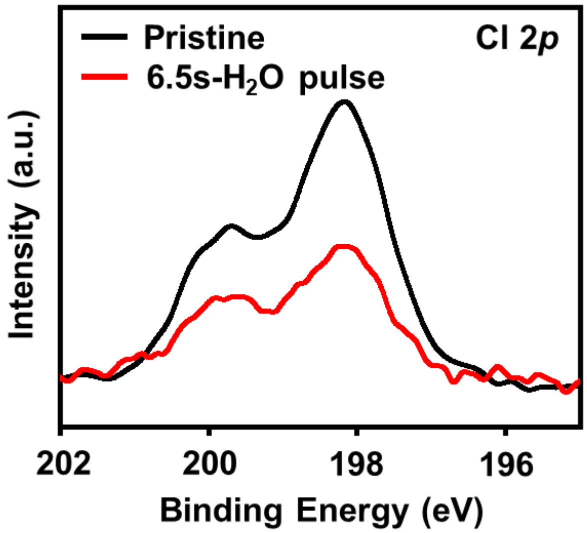

Figure S7. Cl 2*p* XPS core spectra of pristine TiO_2 (H_2O pulse was 0.5 s, black), and TiO_2 deposited using extended H_2O pulse (6.5 s, red).

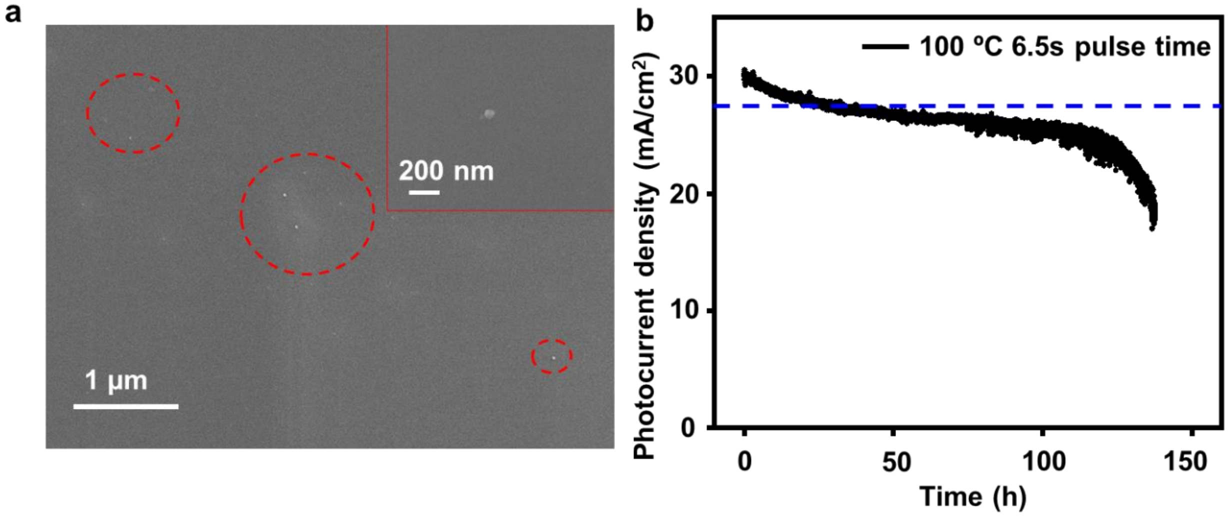

Figure S8. a Top-view SEM of 100 ºC 200cycles Si/TiO_2 grown under elongated water pulse time of 6.5 s during deposition. **b** Chronoamperometry test of Si/TiO_2/Ni under the external bias of 1.8 V vs. RHE at 1 sun illumination. Blue dashed line marks the 90% threshold value.

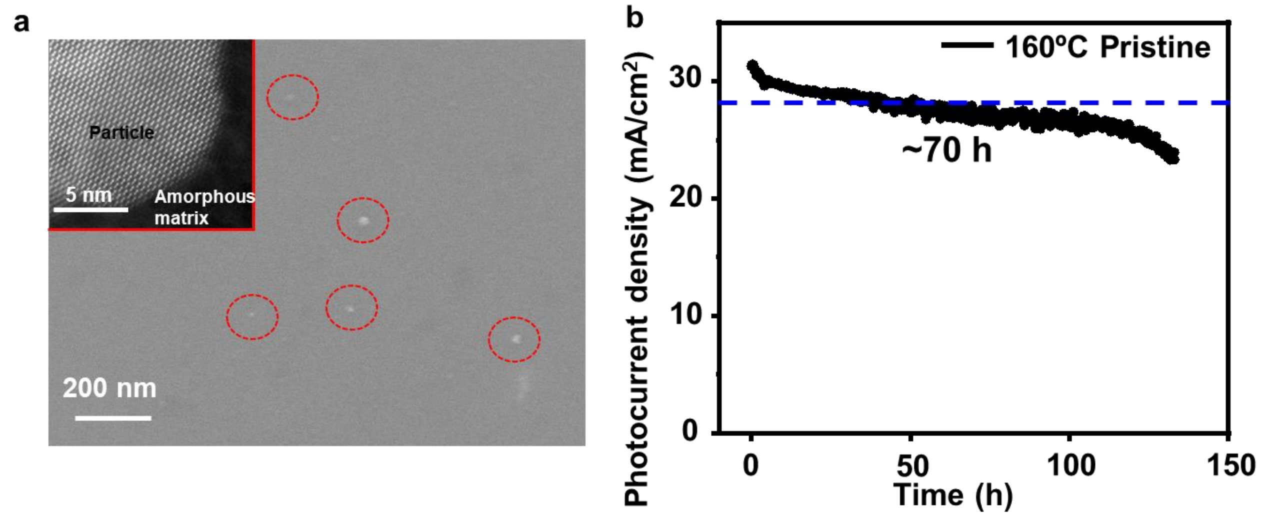

Figure S9. **a** Top-view SEM image of free-standing 160 ºC-grown pristine TiO_2 films. Nanoparticles can be observed from the film surface, marked by red dashed circles. Inset is the HR-STEM image of the nanoparticle showing its crystalline phase. **b** Chronoamperometry test of Si/TiO_2/Ni under the external bias of 1.8V vs. RHE at 1 sun illumination. Blue dashed line marks the 90% threshold value.

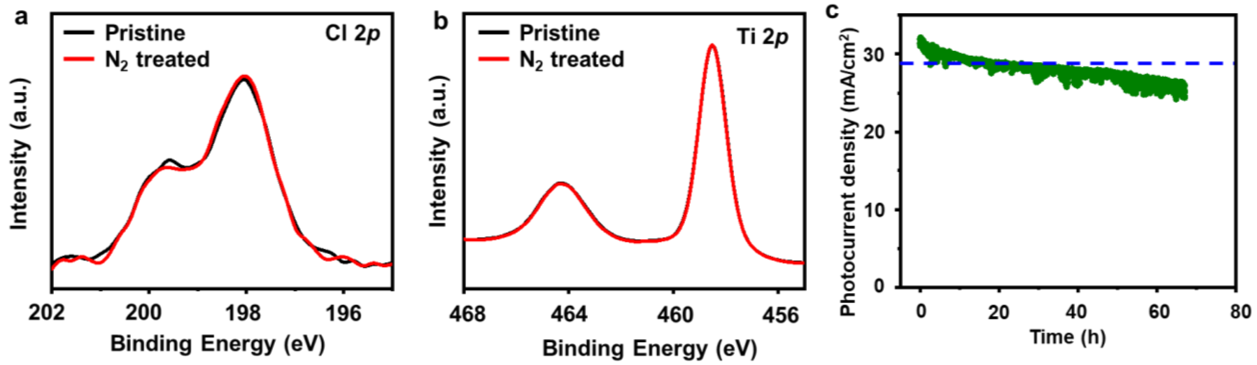

Figure S10. **a** Cl 2*p*, **b** Ti 2*p* XPS core spectra analysis of pristine and N_2 treated TiO_2 protection film. **c,** Chronoamperometry test of Si/TiO_2/Ni with 100 ºC N_2 treated TiO_2 under the external bias of 1.8V vs. RHE at 1 sun illumination. Blue dashed line marks the 90% threshold value.

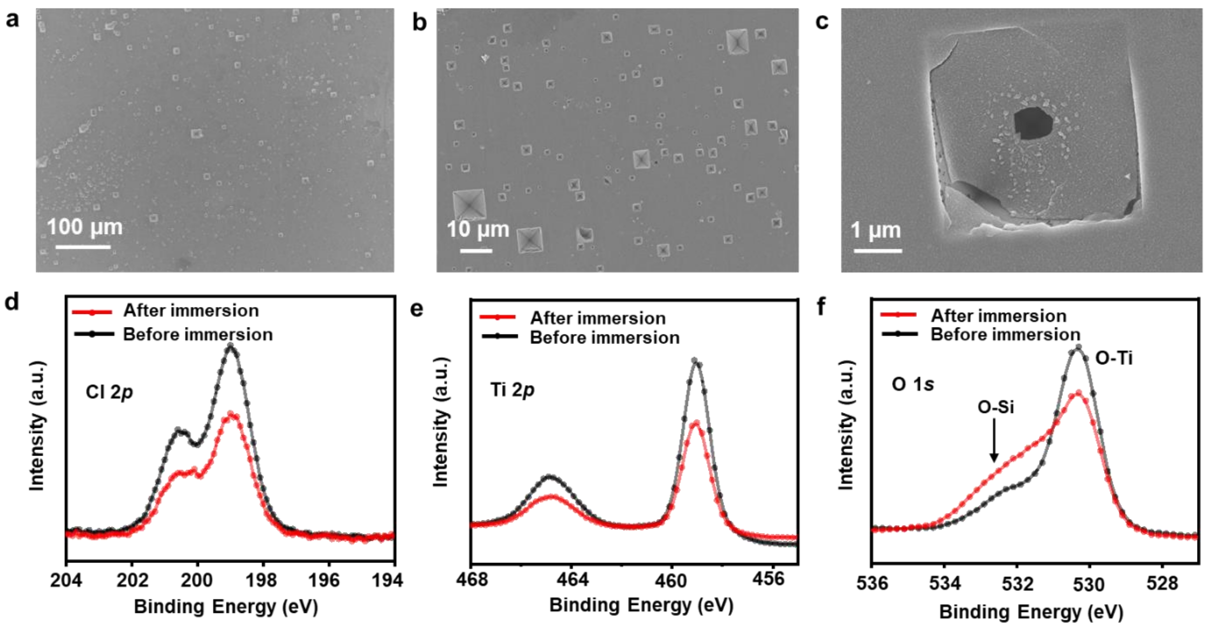

Figure S11. a-c Surface morphology of $TiCl_4$ treated Si/TiO_2 after 1 day immersion in 1M KOH. **d-f** Corresponding XPS core spectrums of $TiCl_4$ treated TiO_2 before and after immersion **(d)** Cl 2*p*, **(e)** Ti 2*p*, **(f)** O 1*s*.

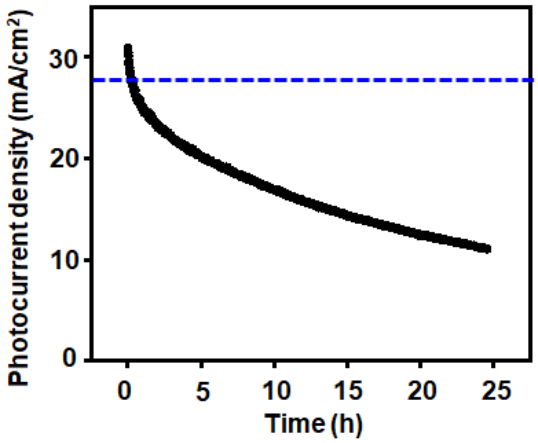

Figure S12. Chronoamperometry test of $Si/TiO_2/Ni$ with 100 ºC $TiCl_4$ treated TiO_2 under the external bias of 1.8V vs. RHE at 1 sun illumination. Blue dashed line marks the 90% threshold value.

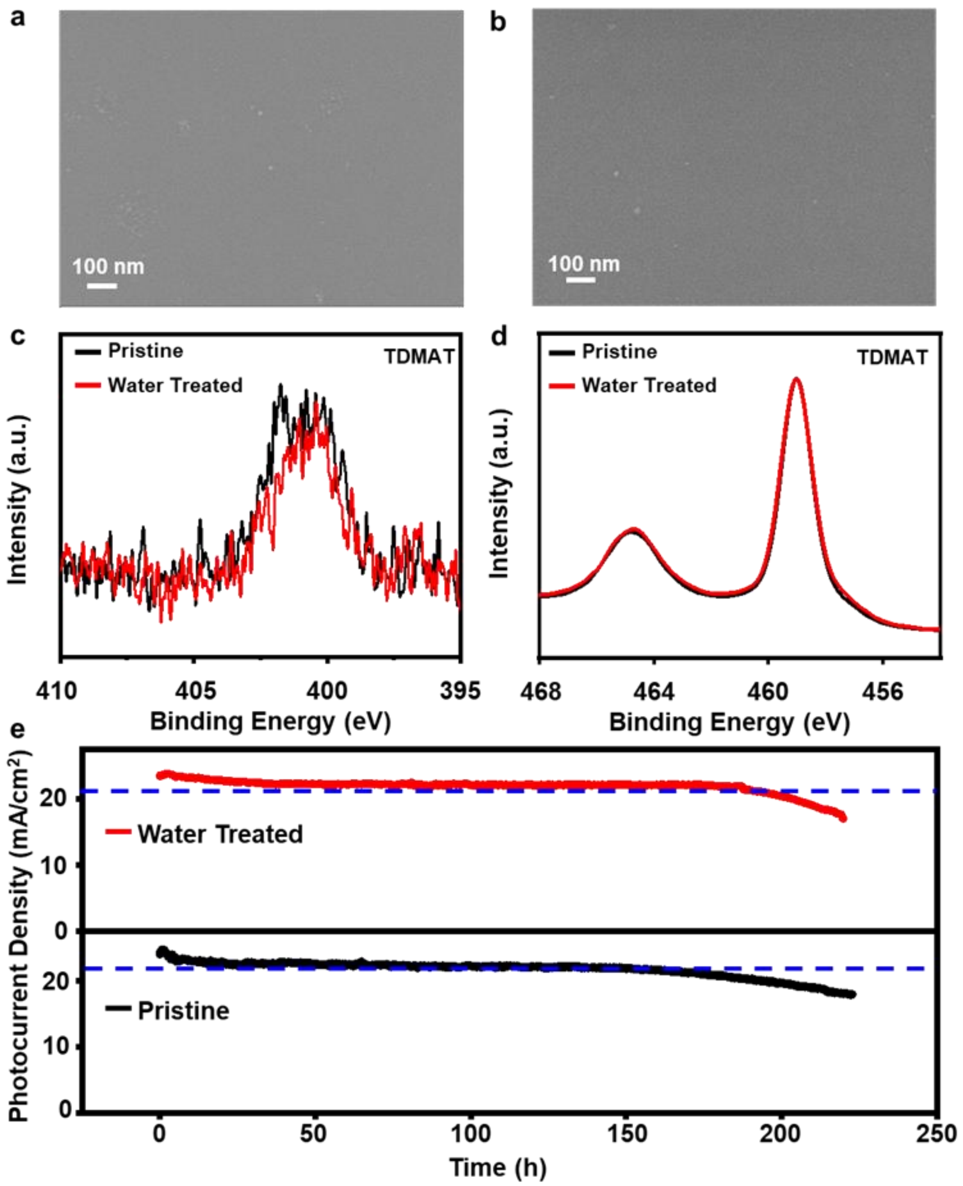

Figure S13. Si photoanode protected by pristine and water treated TiO_2 film from TDMAT precursor. a, **b** Top view SEM images of pristine and water treated TDMAT-TiO_2 film on n-Si substrate. **c** XPS N 1*s* peak of pristine and water treated TiO_2 film from TDMAT. **d** XPS Ti 2*p* peak of pristine and water treated TiO_2 film from TDMAT. **e** Chronoamperometry test of pristine and water treated TDMAT-TiO_2 protected Si photoanode measured in 1.0 M KOH aqueous solution under 1 sun illumination at an external bias of 1.8 V vs. RHE. Blue dashed line marks the 90% threshold value.
